# 

**Published:** 1843-10

**Authors:** 


### BOOKS RECEIVED FOR REVIEW.

1. An Inaugural Lecture on Botany, read at King's College, London, May, 1843. By E. Fisher, F.L.S. &c.—London, 8vo, pp. 27.

2. Brighton and its Three Climates, &c. By A. L. Wigan, M.D.—London, 1843. 8vo, pp. 71. 2s. 6d.

3. Facts and Observations relative to the Influence of Manufactures upon Health and Life. By Daniel Noble, Surgeon.—London, 1843. 8vo, pp. 82.

4. Phreno-Magnetism unmasked. By T. C. Hall, M.D.—Belfast, 1843. 8vo, pp. 20.

5. On Man's power over himself to prevent or control Insanity. By the Rev. J. Barlow, M.A.—Lond. 1843. 12mo, pp. 68.

6. Photographic Manipulation.—Lond. 1843. 8vo, pp. 42.

7. Glyphography, or Engraved Drawing. Second Edition.—Lond. 1843. 8vo, pp 16.

8. The Iodated Waters of Heilbrunn in Bavaria. By Sir A. M. Downie, M.D.—Frankfort, 1843. 12mo, pp. 92.

9. Scarlatina and its treatment on Homœopathic principles. By J. Belluomini. —London, 1843. 8vo, pp. 31.

10. Advice to Wives on the management of themselves during the periods of Pregnancy, Labour, and Suckling. By Pye R. Chavasse, Surgeon. Second Edit. 8vo, pp. 91, 1843.

11. Advice to Mothers on the management of their Offspring. By Pye R. Chavasse. Third Edition.—London, 1843. 8vo, pp. 213.

12. Neurypnology; or the Rationale of Nervous Sleep, considered in relation with Animal Magnetism. By James Braid, Surgeon.—Lond. 1843. 12mo, pp. 265. 5s.

13. A Medical Visit to Graefenberg, for the purpose of investigating the merits of the Water-cure Treatment. By Sir C. Scudamore, M.D.—London, 1843. 8vo, pp. 105. 4s.

14. Letter to Sir Robert Peel on the Responsibility of Monomaniacs for the Crime of Murder. By James Stark, M.D. F.R.S.E.—Edinburgh, 1843. 8vo, pp. 42.

15. Description of the Skeleton of an extinct Gigantic Sloth, &c. By Richard Owen, F.R.S. &c.—London, 1842. 4to, pp. 176.

16. On the Physical causes of the high rate of Mortality in Liverpool. By W. H. Duncan, M.D.—Liverpool, 1843. 8vo, pp. 76.

17. Observations on Idiopathic Dysentery, as it occurs in Europeans in Bengal, particularly in reference to the Anatomy of that Disease. By Walter Raleigh, Surgeon, &c.—Calcutta, 1842. 8vo, pp. 150.

18. Lectures on the Comparative Anatomy and Physiology of the Invertebrate Animals; delivered at the Royal College of Surgeons, in 1843. By Richard Owen, F.R.S. From Notes taken by W. W. Cooper, Surgeon.—London, 1843. 8vo, pp. 392. 14s.

19. Pulmonary Consumption successfully treated with Naphtha. By John Hastings, M.D.—Lond. 1843. 8vo, pp. 120.

### INDEX TO VOL. XVI.

OF THE

### BRITISH AND FOREIGN MEDICAL REVIEW.

PAGE

African fever, Drs. M'William and Pritchett on . . 259-505
Ague, epidemic in Persia . . 558
Alcohol, action of on the blood . 218
on spleen and liver 219
Allantois . . . . 556
Allnatt, Dr., on neuralgia . . 511
Amnion, chorion, &c. . . 550
Amputation, history of . . 63
Aneurism, history of operations for . 62
Antidotes, Dr. Paris on . . 195
Antilithics . . . 196
Antiseptics, Dr. Paris on . . ib.
Antispasmodics . . . . 192
Aorta, on disease of . . 328
Apoplexy, M. Gay on . . 272
Army, health of the United States . 34
Arnold, Dr., on the reflex theory . 158
Arsenic, new test for . . 275
Ashwell, Dr., on female diseases . 512
Assimilation, cultivation of . 217
Dr. Prout on . 481
Hilton on . . 322

Babington, Dr., on gall-bladder . 320
Barlow, Dr., on disease of the kidney 308
early youth 326
Barry, Dr., criticism of his views . 522
Becquerel, M., on urinary semeiology 328
Bell, Dr. W., on epidemic ague . 558
Bengal Dispensatory . . 352
Bird, Dr., on urinary globules . 321
deposits . 312
Bischoff, Dr., on medicine and surgery 507
oval development . 513
Birnbaum, Dr., on changes in the cervix uteri . . 184
Blood, Dr. Schultz on the composition of . . . 210
Blood, on the formation of . 224
ripening of . . . 226
hemorrhoidal, nature of . 227
menstrual, nature of . 228
its influence in muscular spasm 418

PAGE

Blood-corpuscles in the camelidæ and deer . . . 287
Blood-vesicles, origin of . . 212
Blood-stains, jurisprudence of . 74
Bones, exhumation of . · 71
regeneration of . . 504
Books for review . . 287, 566
Bougard, Dr., on delirium tremens . 506
Brain, treatise on softening of . 1
diseases of, in United States . 43
Brighton, its climate . . 500
Bronchitis, Dr. Graves on . 244
Bulard, M., on the plague . 289

Calcutta, medical topography of . 206
Camphor, virtues of . . 360
Canstatt, Dr., his annual report . 268
Carbonic acid exhaled from the lungs 285
Cartilage, regeneration of . . 504
Cataract, on the operation for . 326
Catheterism, history of . . 56
Cervix uteri, on changes in . 184
Chemistry, its relation to medicine . 145
Chevers, Dr., on the aorta . 323
Chimpansé, M. Vrolik on . 373
Cholera, new species of . . 558
Crichton, Sir A., on criminal insanity 81
Chylification, culture of . . 223
Clay, Dr., on extirpation of ovaria . 387
Climate of the United States . 34
of Brighton . . 500
Cholera in the United States army . 43
Chorion, development of . 550
Clinical medicine, Dr. Graves on . 232
Clot-Bey, M., on the plague . 289
Cock on injuries of the head . 312
Combustion, spontaneous . . 74
Consumption, M. Louis' treatise on . 425
Dr. John Hastings on 490
morbid anatomy of . 425
symptomatology of . 438
Cooper, Sir A., life of . . 118
Mr. B., his life of Sir A. Cooper . . . ib.

PAGE
Copeman, Mr., on apoplexy . 272
Corpora lutea . . . 526
Crises, pathology of . . 216
Cystic oxide . . . 315

Davy, Dr., on quarantine . . 289
Delirium tremens, thesis on . 506
Digestion, new experiments on . 221
Dr. Prout on . . 481
Digestive process, culture of . 220
Diaphoretics, Dr. Paris on . . 194
Diluents, Dr. Paris on . . 199
Diuretics, Dr. Paris on . . 193
Durand-Fardel, M., on softening of the brain . . . 1

Ebriety in the United States army . 43
Effects of Indian hemp . . 357
Emetics, Dr. Paris on . . 192
Emmenagogues, Dr. Paris on . ib.
Emmert, Dr., on pathology . 267
Enlargement of liver . . 253
Exostosis, account of . . 382
Expectorants, Dr. Paris on . 194
Eye, Dr. Hall's remarks on . 270
internal inflammation of . 323

Factories, Mr. Noble on the health of 507
Fainting fever of Persia . . 558
Fecundation, history of . . 520
Female diseases, Dr. Ashwell on . 512
Fever, Dr. Graves on . . 233
African, Dr. M'William on . 259
Dr. Pritchett on . 505
Fevers, their prevalence in the United States . . . 39
Flourens, M., on the nervous system 364
Follicles, Graafian . . . 513
Forry, Dr., on United States climate 34
Fracture of the thigh-bone, new mode of treating . . . 508

Germinal vesicle . . . 517
Glands, Cowper's, in the female . 155
Gout, Dr. Todd on . . 460
the materies morbi of . 467
Graafian follicles . . 513
formation of . 518
Graves, Dr., his clinical medicine . 232
Guy's Hospital Reports, vol. vii. . 308

Hemoptysis, Dr. Graves on . 248
Hall, Dr., on diseases of the nervous system . . 158
exposition of his reflex theory . . ib.
Hall, Dr. John, on the eye . 270
Harrisson, Mr., on spinal deformity . 341
Harelip, history of the operation for 50
Hastings, Dr. John, on naphtha . 490
Head, injuries of . . 312

PAGE
Hebra, Dr., his history of surgical operations . . 46
Heine, Dr., on spontaneous dislocations . . . 486
Hemorrhage after delivery . 320
Hemp, Indian, effects of . . 357
Hernia, history of operations for . 57
Mr. Key on . . 319
History of the operation for harelip . 50
operations for hernia . 57
laryngotomy . . 51
lithotomy . . 58
Hip, on spontaneous dislocations of . 486
Huenefeld, Dr., on chemistry and medicine . . . 145
Hughes, Dr., on the location of phthisis 317
pneumonia . 320
Human life, on the renewal of . 208
Hygiène, mental, Dr. Sweetser on . 511

Infanticide, jurisprudence of . . 68
Mr. Taylor on . 309
Inflammation, Dr. Graves on . 257
Dr. Klencke on . 503
Influenza, Dr. Graves on . . 243
Irideremia . . . 320
Insanity, on the plea of in criminal cases . . . 81
Intestinal canal, moulting of . 223

Jones, Mr. Wharton, on the ovum . 513
Jurisprudence, Nicolai's manual of . 68

Key, Mr., on hernia . . 319
Kidney, on the diagnosis of diseases of 308
King, Mr., on digestion of the stomach 310
Klencke, Dr., on inflammation . 503

Languor, Dr. Wilson on . . 418
Laryngitis, Dr. Graves on . . 247
Laryngotomy, history of . . 51
Lever, Mr., on pelvic tumours . 310
uterine hemorrhage . 320
Life, human, on the renewal of . 208
Life of a travelling physician . . 285
Lithotomy, history of . . 58
Liver, enlargement of . . 253
Loudon, Dr., on population . 229
Louis, M., on consumption . 425
Lunacy, on the law of . . 81
Lungs, diseases of in North America 42
on the weight of . . 309
artificial inflation of . 310

M'Naughten, trial of . . 81
M'William, Dr., on the Niger Fever . 259
Malapraxis, jurisprudence of . 72
Magnetism and electricity, cures by . 269
Man, on his rejuvenescence . 208
Martin, Mr., on the health of Calcutta 206
Malta, history of plague at . 294

LIBRARY OF THE

Orleans Parish Medical Society

PAGE
Manufactures, influence of on health 507
Marx, Dr., on pathology and therapeutics . . . 473
Materia Medica, Dr. Thomson's . 359
Dr. Pereira's . 362
Medicine, its relation to chemistry . 145
Medicine and surgery, their relations 507
Mojsisovics, Dr., on fractures of the thigh . . . 508
Morbid anatomy, treatises on . 381
Morgan, Mr., on cataract . . 326
Moulinié, Dr., on surgery . 266
Moulting, physiology of . . 209
Muscles and nerves, renewal of . 214
Muscular system, Dr. Wilson on . 418

Naphtha, alleged specific in phthisis . 490
Nerves, regeneration of . . 503
Nervous system, Dr. Hall on the diseases of . . . 158
Nervous system, M. Flourens on . 364
Neuralgia, Dr. Allnatt on . . 511
Nicolai, Dr., his jurisprudence . 68
Niger, Dr. M'William on the fever of 259
Noble, Mr., on the health of factories 507

Œsophagotomy, history of . . 53
Opium, on the mode of preparing . 354
Opium eating . . . 355
smoking . . . 356
O'Shaughnessy, Dr., his dispensatory 352
Ovaria, on the extirpation of . 387
Ovarian tumours, cases of . 388
Ovum, Dr. Bischoff on . . 513
development of . . 542
of the mammifera, report on . 513

Paraplegia, Dr. Graves on . . 254
Paris, Dr., his pharmacologia . 188
Plasma, nature and origin of . 213
Patriarchs, on the lives of . . 510
Pelvic tumours, Mr. Lever on . 310
Persia, new epidemic in . . 558
Pharmacologia, Dr. Paris's . 188
Philological delinquencies . . 492
Phthisis, Dr. Graves on . . 249
on the location of . 317
M. Louis on . . . 425
morbid anatomy of . ib.
natural history of . . 438
diagnosis of . . 450
causes of . . . 452
treatment of . . 454
how is it propagated . 291
Physician, life of a travelling . . 285
Plague, treatises on . . 289
Pleuritis, curious case of . . 322
Ploucquet's test . . . 309
Pneumonia, Dr. Hughes on . 320
Poisoning by arsenic, case of . 322
jurisprudence of . . 75

PAGE
Population and subsistence, Dr. Loudon on . . . 229
Paracentesis thoracis, history of . 53
abdominis, history of . 55
Prescribing, the theory and art of . 197
Prichard, Dr., on criminal insanity . 81
Pritchett, Dr., on the African fever . 505
Prout, Dr., on stomach and renal diseases . . . 477
Prout, Dr., his peculiar views . 485
Purgatives, Dr. Paris on . . 192

Quarantine, treatises on . . 289
laws, Mr. Holroyd on . 290

Reflex-function, doctrine of . 158
Refrigerants, Dr. Paris on . . 1[illegible]5
Reinsch, M., his new test for arsenic 275
Rejuvenescence, on human . . 208
Renal diseases, Dr. Prout on . 477
Report on arsenic . . 275
on the ovum . . 513
on the Persian ague . 558
Review, British and Foreign . 490
Rheumatism, Dr. Todd on . 460
Rieken, Dr., on scarlatina . . 511
Rumball on insanity . . 81

Saliva, on the digestive powers of . 222
Sampson, Mr., on criminal insanity . 81
Scarlatina, treated by ammonia . 512
Scarlet fever, Dr. Graves on . 241
Schultz, Dr., on the renewal of human life . . . . . 208
Scorbutus in the United States army . 44
Sedatives, Dr. Paris on . . 191
Smith on origin of disease . . 510
Softening of the brain, treatise on . 1
acute . ib.
chronic . 18
Spasm, Dr. Wilson on . . 418
Spinal system, true, Dr. Hall's . 167
physiology of . 168
pathology of . 169
Spine, on deformities of . 341-7
Stomach and œsophagus, digestion of 310
Stomach diseases, Dr. Prout on . 477
Stimulants, Dr. Paris on . . 189
Subsistence, on the problem of . 229
Suicide, medical jurisprudence of . 73
Superbiam sume . . . 490
Surgery, on success in . . 266
Surgical operations, history of . 46
Sweetser, Dr., on mental hygiène . 511

Tavernier, Dr., on spinal deformity . 347
Taylor, Mr., his report on arsenic . 275
on infanticide . 309
Teheran, epidemic at . . 558
population of . ib.
Tiedemann, M., on Cowper's glands . 155

PAGE
Todd, Dr., on gout and rheumatism . 460
Tracheotomy, history of . . 51
Trephining, history of . . 47
Tic douloureux, Dr. Allnatt on . 511

Umbilical vesicle . . 555
United States, climate of . . 34
Uric acid . . . 313
oxide . . . 315
Urinary deposits, Dr. Budd on . 312
globules, microscopic . 321
Urine, changes of in disease . . 328
chemical and physical poperties of . . . 328
Urine, in fever . . . 334
in anemia . . 335
alkaline . . . 336
in scarlatina . . 337
in diseases of the spinal marrow 339

PAGE
Valetta, view of . . 295
Van Helmont's system of medicine . 406
account of his life . 403
psychology . . 411
pathology . . 413
therapeutics . . 410
Vrolik, Professor, on the chimpansé . 373

Water-cure, physiology of . 228
Wetzler, Dr., on magneto-electricism 269
Wigan, Dr., on Brighton climates . 500
Wilson, Dr., on spasm, languor, &c. 418
Winslow, M., on criminal insanity . 81
Women, Dr. Ashwell on diseases of 512

Youth, on diseases in early . 326

Zona pellucida . . . 515

END OF VOL. XVI.

C. AND J. ADLARD, PRINTERS, BARTHOLOMEW CLOSE.

